# Upregulation of Leukotriene Receptors in Gastric Cancer

**DOI:** 10.3390/cancers3033156

**Published:** 2011-08-08

**Authors:** Marino Venerito, Doerthe Kuester, Caroline Harms, Daniel Schubert, Thomas Wex, Peter Malfertheiner

**Affiliations:** 1 Department of Gastroenterology, Hepatology and Infectious Diseases, Otto-von-Guericke University, Leipziger Str. 44, Magdeburg 39120, Germany; E-Mails: m.venerito@med.ovgu.de (M.V.); caroline.harms@web.de (C.H.); peter.malfertheiner@med.ovgu.de (P.M.); 2 Institute of Pathology, Otto-von-Guericke University, Leipziger Str. 44, Magdeburg 39120, Germany; E-Mail: doerthe.kuester@med.ovgu.de; 3 Department of General, Visceral and Vascular Surgery, Otto-von-Guericke University Magdeburg, Leipziger Str. 44, Magdeburg 39120, Germany; E-Mail: daniel.schubert@med.ovgu.de

**Keywords:** leukotriene receptors, gastric cancer, cysteinyl-leukotrienes

## Abstract

**Background:**

Leukotrienes (LT) mediate allergic and inflammatory processes. Previously, we identified significant changes in the expression pattern of LT receptors in the gastric mucosa after eradication of *Helicobacter pylori* infection. The aim of the present study was to evaluate the expression of 5-lipoxygenase (5-LOX) and LT receptors in gastric cancer (GC).

**Methods:**

The expression of 5-LOX and receptors for LTB4 (BLT-1, BLT-2) and cysteinyl-LT (CysLT-1, CysLT-2) were analyzed by immunohistochemistry (IHC) in GC samples of 35 consecutive patients who underwent gastrectomy and in 29 tumor-free tissue specimens from gastric mucosa.

**Results:**

Male-to-female ratio was 24:11. The median age was 70 years (range 34–91). Twenty-two patients had GC of intestinal, six of diffuse, six of mixed and one of undifferentiated type. The IHC analysis showed a nearly ubiquitous expression of studied proteins in GC (88–97%) and in tumor-free specimens as well (89–100%). An increase in the immunoreactive score of both BLT receptors and CysLT-1 was observed in GC compared to tumor-free gastric mucosa (p < 0.001 for BLT-1; p < 0.01 for BLT-2 and CysLT-1, Mann-Whitney U-test). No differences in the IHC expression of 5-LOX and CsyLT-2 were observed between GC and tumor-free mucosa. The expression of BLT-2, CysLT-1 and CysLT-2 was increased in GC of intestinal type when compared to the diffuse type (p < 0.05; Mann-Whitney U-test).

**Conclusions:**

LTB4 receptors and CysLT-1 are up-regulated in GC tissue implying a role in gastric carcinogenesis.

## 1. Introduction

Gastric cancer (GC) incidence has been rapidly declining in most Western countries [1]. For instance, in Germany, the standardised mortality rate has declined by more than 80% in the last six decades; 24,600 and 8,200 individuals died from GC in former West Germany in 1952 and 2007, respectively [2]. Similar changes in the incidence rates have been reported for most other European countries, with a reduction of up to 5.4% per year between 1994 and 2005 [3]. The decrease of GC incidence has been steady and well documented for decades [3-5], and reflects most likely the reduction of risk factors for GC, in particular changes in food preservation (cooling and freezing instead of salting, smoking and fermentation) and a decreasing prevalence of *Helicobacter pylori (H. pylori)* by birth cohort [3]. Despite this reduction in several regions, GC remains a clinical challenge and burden worldwide, individually and socio-economically. Due to the predicted growth of the world population and the increased life expectancies in most countries, the absolute number of GC cases is likely to stabilize or even to increase in the future reaching up to 900,000 new GC cases each year, with an annual associated death toll of 700,000 [1]. Regarding the clinical management of GC, the five year survival rate has not improved significantly and is still below 30% in most countries, except Japan. The high mortality rate in GC is mostly due to its detection in advanced stage, when clinical or alarm symptoms become apparent. Based on the stage at diagnosis, different therapeutic regimens including surgery and neoadjuvant or adjuvant chemotherapy are currently used [6-9].

Leukotrienes and lipoxines belong to the large group of eicosanoids that originate from the oxidative degradation of arachidonic acids [10]. Numerous studies have shown the pleiotrope effects of eicosanoids on various cellular functions and their role in pathologies including chronic inflammation and cancer [10-12]. The role of chronic inflammation as “trigger” for carcinogenesis is well established also for GC. The strong association of GC with the *H. pylori* infection has led to the classification of this bacterium as “definite carcinogen” (class I) by the World Health Organization in 1994 [13]. This classification was reconfirmed in 2009 after intensive re-evaluation of data published between 1994 and 2008 [14]. Despite the fact that only 10–15% of *H. pylori*-infected subjects present symptoms and/or clinical diseases, all infected individuals present gastric inflammation (gastritis) histologically [15]. The presence of gastritis in humans as well as in animal models is associated with molecular alterations such as elevated cytokine expression, increased generation of eicosanoids by cyclooxygenases and lipoxygenases and upregulation/activation of the respective receptors for these mediators [15-18]. Recently, we studied the expression profile of various eicosanoid-related enzymes (COX-1, COX-2, 5-LOX) and -receptors (BLT-1, CysLTR1) in *H. pylori* infection, and identified significant changes in the expression of these molecules in context to this infection [18]. While the central role of COX-2 in carcinogenesis of epithelial tumors is well established [19], similar knowledge for 5-LOX and leukotriene receptors does not exist. Taking into consideration recent studies demonstrating an involvement of leukotriene receptors in pancreatic, colon, urinary bladder and breast cancer [12,20-23], and their identification in *H. pylori*-induced gastritis [18], we hypothesized that the expression of these receptors might be deregulated in GC. Therefore, the expression pattern of the two leukotriene B4 receptors BLT-1 and BLT-2 as well as the two receptors for cysteinyl-leukotrienes (CysLT-1 and CysLT-2) was studied by immunohistochemistry in a prospective study cohort of patients with GC. Since the ligands for all these four receptors are generated via the 5-LOX pathway, the expression of the corresponding enzyme, 5-LOX, was analyzed as well.

## 2. Results

### 2.1. Expression of LT-Receptors in Gastric Mucosa

**Figure 1.**
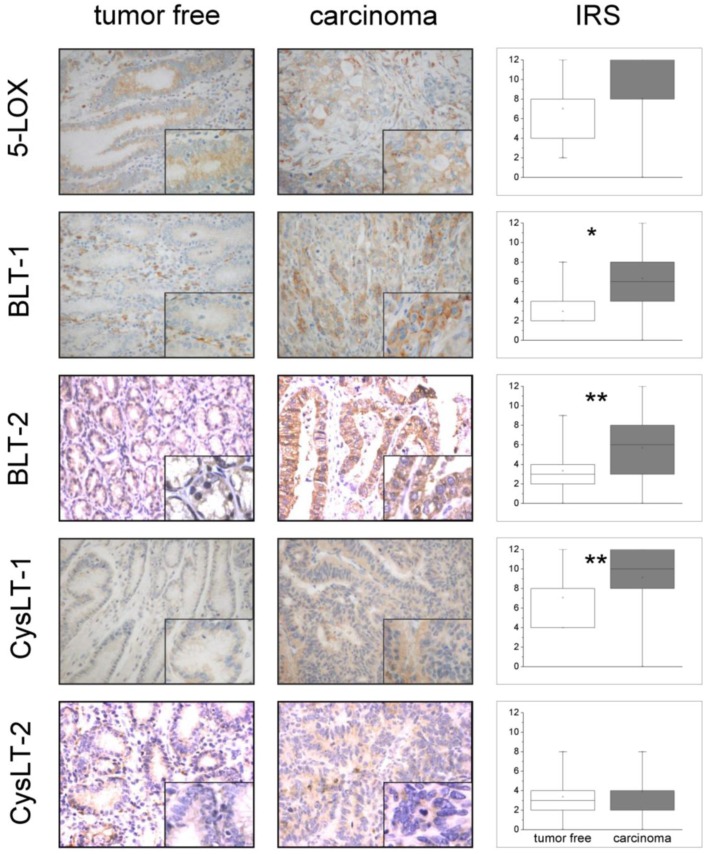
Immunohistochemical (IHC) expression of 5-LOX, BLT-1, BLT-2, CysLT-1, CysLT-2 in GC and tumor-free gastric mucosa. *On the left side* of the figure are shown IHC staining patterns from tumor-free gastric mucosa and GC specimens, respectively. Each slide displays a representative section of the gastric surface epithelium at low (whole image) and high magnification (right lower corner). Signals illustrating 5-LOX, BLT-1, BLT-2, CysLT-1, CysLT-2 appear in brown (microscope: Nikon F200 camera, Nikon coolpix 990; 100×, 400×). *On the right side* of the figure is shown the IHC expression of 5-LOX and LT-receptors in the tumor-free gastric mucosa and GC, respectively. Values are presented as box plot graphics where each box plot shows the location of the median (marked line), mean (open square) and the 25^th^ and 75^th^ quartiles (inferior and superior limit of boxes). IRS: immunoreactive score. * denotes p < 0.01 (BLT-1), ** denotes p < 0.001 (BLT-2 and CysLT-1) (Mann-Whitney U-test).

Figure 1 illustrates representative immunohistochemical staining patterns of antibodies against 5-LOX, BLT-1, BLT-2, CysLT-1 and CysLT-2. In general, the gastric surface and foveolar epithelium showed a medium to strong cytoplasmatic immunostaining for 5-LOX and CysLT-1 and a weak to medium expression pattern for BLT-1, BLT-2 and CysLT-2. A medium to strong cytoplasmatic positivity was seen in parietal cells for all LT-receptors, whereas 5- LOX was expressed weakly and infrequently in corpus glands. Among immune cells, plasma cells showed the strongest reactivity for both LTB4-receptors and 5-LOX and a medium reactivity for both CysLT-receptors. Granulocytes showed a strong cytoplasmatic positivity for CysLT-1 and CysLT-2 but only a weak positivity for 5-LOX and LTB4-receptors. For lymphocytes, a strong cytoplasmatic expression was observed for CysLT-2 whereas all other antibodies showed a slight to medium staining intensity.

### 2.2. Upregulation of LT-Receptor Expression in GC

Immunohistochemical analysis showed a nearly ubiquitous expression of studied proteins in GC (88–97%) and in tumor-free specimens as well (89–100%). No differences in the percentage of specimens expressing the five proteins studied were observed between GC and tumor-free specimens (data not shown). The detailed proportion of GC specimens expressing 5-LOX and LT-receptors are shown in Table 1. IHC expression of 5-LOX and LT-receptors in GC and tumor-free surface epithelium are shown in Figure 1. A slightly increased expression of 5-LOX in GC specimens compared to tumor-free surface epithelium was observed, but this difference was not statistically significant. A significant increase in the immunoreactive score of both BLT receptors and CysLT-1 was observed in GC compared to tumor-free gastric mucosa (p < 0.001 for BLT-1; p < 0.01 BLT-2 and CysLT-1, Mann-Whitney U-test). No differences in the expression of CysLT-2 between GC specimens and tumor-free surface epithelium were observed.

### 2.3. 5-LOX and LT-Receptor Expression in GC of Intestinal Type vs. Diffuse Type

**Figure 2.**
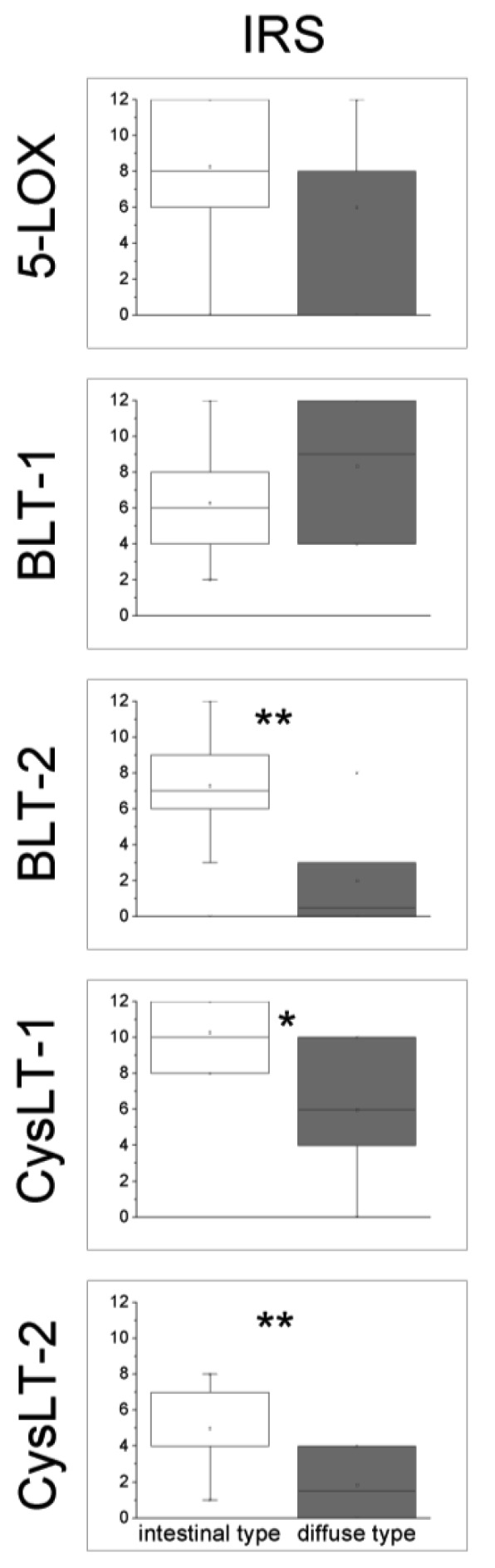
Immunohistochemical (IHC) expression scores of 5-LOX and LT-receptors in GC of intestinal and diffuse type. Values are presented as box plot graphics for IHC expression of 5-LOX and LT receptors BLT-1, BLT-2, CysLT-1, CysLT-2 in GC of intestinal and diffuse type, respectively. Each box plot shows the location of the median (marked line), mean (open square) and the 25^th^ and 75^th^ quartiles (inferior and superior limit of boxes). IRS: immunoreactive score. * denotes p < 0.05, ** denotes p < 0.01 (Mann-Whitney U-test).

The expression scores of 5-LOX and LT-receptors in GC of intestinal type and diffuse type are shown in Figure 2. No differences in the expression of 5-LOX and BLT-1 were observed between the two histological types of GC, whereas the expression of BLT-2 was increased in GC specimens of intestinal type compared to the diffuse type (p < 0.01, Mann-Whitney U-test). GC specimens of intestinal type showed also an increased immunoreactive score for both CysLT-receptor antibodies when compared to the diffuse type (p < 0.05 for CysLT-1 and p < 0.01 for CysLT-2, respectively).

### 2.4. 5-LOX and LT-Receptor Expression according to Tumor Stage

Post-operative tumor grading and staging given by pathologic examination of surgical specimens are shown in Table 2. Because of the small number of patients in each group, a statistical analysis of the influence of tumor stage on IHC expression of 5-LOX and LT-receptors was possible only for the subgroup of gastric cancer of intestinal type. In this group, no differences in the expression of 5-LOX and LT-receptors were observed when stage I plus II and stage III plus IV were compared (p > 0.05, Mann-Whitney U-test).

## 3. Discussion

In this study, the expression of 5-LOX and LT-receptors in GC tissue was studied by IHC. The 5-LOX/LT-receptor system is involved mostly in inflammatory and allergic processes and only recently its expression has been demonstrated in a number of cancers like pancreatic, colon, urinary bladder and breast cancer [12,20-23]. Herein, we show that 5-LOX and LT-receptors are expressed also in GC. To our knowledge, this is the first study that reports the expression of 5-LOX and LT-receptors in GC.

The inhibition of 5-LOX by MK886 has been shown to augment the antitumor activity of celecoxib in human colorectal cancer [24]. Furthermore, in a study on 120 colonic adenomas, a significant correlation between high 5-LOX expression and clinical predictors for malignant transformation were found [25]. In our study, no differences in the IHC expression of 5-LOX were observed between GC and tumor-free mucosa. However, the present study was not conceived to evaluate either the activity of 5-LOX, or the expression/activity of the 5-LOX activating protein (FLAP), which is essential for 5-LOX activity [26]. Thus, on the basis of the current knowledge, a possible role of 5-LOX as a gene candidate for prevention or treatment of GC cannot be ruled out.

Compared to tumor-free gastric mucosa we found an up-regulation of the expression of BLT-1 in GC tissue. In a previous study conducted on patients with *H. pylori*-induced corpus predominant gastritis, successful eradication of *H. pylori* resulted in an increased expression of BLT-1 in gastric surface epithelium [18]. The increased expression of BLT-1 both after *H. pylori* eradication and in GC suggests a potential role of the receptor in cell proliferation and renewal. Studies on colon cancer support this hypothesis. Indeed, an increased expression of BLT-1 has been shown in colon-cancer tissue and cell lines [27], and the suppression of BLT-1 in cultured cells by a small interfering RNA turned out in a decrease of cell proliferation [28]. In another study on six human pancreatic cancer cell lines, LTB4 was shown to directly regulate the growth of pancreatic cancer cells, whereas the blockade of BLT-1 by the BLT-1 antagonist LY293111 caused both time- and concentration-dependent inhibition of proliferation of all six cell lines studied [29]. However, a randomized double-blind phase II trial revealed that combining BLT-1 antagonist LY293111 and gemcitabine did not add any benefit in terms of survival to chemotherapy-naïve patients with advanced pancreatic carcinoma [30]. Whether the use of a BLT-1 antagonist might be an option for treating GC has not been studied.

In addition to BLT-1, BLT-2 was also found to be up-regulated in GC tissue. Recent studies suggest that BLT-2 may have a role in cancer spread, and thus influence prognosis in patients with malignancies. In a mouse model, the pretreatment with the BLT-2 inhibitor LY255283 dramatically suppressed the metastatic potential of Ras-transformed cells injected in the tail-vein, whereas the injection of cells overexpressing BLT-2 produced numerous metastatic lung nodules via elevation of MMP-9 activity [31]. In a study on 245 patients with epithelial ovarian cancer, a strong expression of BLT-2 was found in 45% of samples and correlated with worse clinical parameters like advanced stage disease and platinum resistance [32]. This is not the case in GC, as in our study no GC samples showed strong BLT-2 expression, whereas about 90% of GC samples showed a low to intermediate expression of BLT-2.

We identified an increased expression of BLT-2 in GC of intestinal type compared to diffuse type. Indeed, expression of BLT-2 in GC of diffuse type was found to be low to absent. A possible explanation for this observation is that the BLT-2 signal pathway is mainly involved in inflammatory processes, and GC of intestinal type rises in a gastric mucosa with atrophic changes due to a long lasting *H. pylori*-induced inflammation. Thus, the increased expression of BLT-2 in GC of intestinal type may be an early phenomenon in the *H. pylori*-induced gastric carcinogenesis.

The downstream signaling pathway of BLT-2 may support cyclooxygenase-2 (COX-2) in promoting gastric carcinogenesis. COX-2 is overexpressed in GC, whereas knockdown of COX-2 or administration of COX-2 inhibitors suppresses tumor formation in models of GC [33]. In a study on human synovial fibroblasts, LTB4 potently up-regulated and stabilized interleukin-1β-induced COX-2 mRNA and protein expression by activating BLT-2 [34]. Whether the blockade of the BLT-2 alone or in combination with COX-2 inhibitors is applicable for prevention or treatment of GC has not been yet investigated.

Compared to tumor-free gastric mucosa we identified an up-regulation of CysLT-1 in GC. Previously, we described an increased expression of CysLT-1 also in *H. pylori*-induced gastritis, whereas a successful eradication of the bacterium led to a decreased expression of CysLT-1 [18]. Both CysLT-receptors showed an increased expression in GC of intestinal type compared to the diffuse type. An *in vitro* study on four different GC cell lines (AGS, MKN-28, MKN-45, NCI-N87) demonstrated that *H. pylori* infection strongly up-regulated CysLT-2 gene expression [35]. However, functional studies on AGS and NCI-N87 cells revealed that neither the stimulation of both cell lines with LTB4 nor blocking CysLT-2 with BAY-u9773, a non-selective CysLT-receptor antagonist, affected cellular proliferation [Venerito et al., unpublished data]. The missing functional relevance of CysLT-2 in these cell lines and the unchanged expression of CysLT-2 in GC compared to tumor-free gastric mucosa suggest that this receptor has no or only a limited role in gastric carcinogenesis. Further studies, in particular for the CysLT-1 are needed to define its precise role in GC.

Our study presents some limitations. First, the number of patients with GC of diffuse type was small. A study with a larger number of patients with both histological types of GC would strengthen our results. However, the IHC expression of BLT-2 and CysLT-2 in GC of diffuse type was found to be homogeneously low or absent in all specimens suggesting that increasing the number of patients to further investigate the expression of these two receptors in GC would not add much information to the results of the present study.

Second, a correlation of the IHC expression of 5-LOX and LT-receptors in GC tissue of patients studied with their outcome may eventually provide information on the prognosis depending on immunohistochemical score. As most patients received follow-up investigations and eventually further therapy in out-patient departments outside of our institution, this type of investigation was not possible.

Strength of the present study is that all patients received gastrectomy for curative surgical therapy without neoadjuvant therapy. Thus, the IHC expression of 5-LOX and LT-receptors described in the present study concerns GC without previous treatment with chemotherapeutic agents. Further studies are needed to clarify whether treatment with neoadjuvant chemotherapy may influence the expression pattern of 5-LOX and LT-receptors in GC.

## 4. Experimental

### 4.1. Patients and Clinical Samples

Thirty-five consecutive patients with gastric carcinoma who underwent gastrectomy for curative surgical therapy at the Otto-von-Guericke University Hospital of Magdeburg, Germany between March 2005 and August 2007 were selected for this study. All patients received D2 or greater lymph node (LN) dissection. The mean number of total lymph nodes found was 24.2 (range, 8–65; median, 21), and the mean number of metastatic nodes was 8.5 (range, 0–40; median, 6). Male-to-female ratio was 24:11. The median age was 70 years (range 34–91). According to the Lauren classification, 22 (62.8%) gastric carcinomas were of intestinal type, six (17.1%) of diffuse type, six (17.1%) of mixed type and one (2.8%) undifferentiated. Eleven patients had a carcinoma of the cardia region, 12 had a carcinoma of the corpus region and 12 had a carcinoma of the antrum. All patients received primary surgical resection without neoadjuvant treatment. Although all patients received gastrectomy for curative surgical therapy, staging given by pathologic examination after surgery showed in most cases an advanced stage of disease. *Helicobacter pylori* status investigated by serology (IgG against *H. pylori* and anti-CagA antibodies tested by specific EIA) was available in 16/35 patients. Current *H. pylori* infection was diagnosed in 13 out of these 16 patients. Tumor-free tissue samples from gastric antrum and corpus could be retrieved only from 29 patients with GC. The study was approved by the local Ethics committee of the Otto-von-Guericke University.

### 4.2. Tissue Microarrays

All Hematoxylin and Eosin stained slides were reviewed and four representative tumor tissue samples were selected for each case. The corresponding formalin-fixed paraffin-embedded tissue blocks were retrieved. The selected area was circled on the slide with a marker pen for tissue microarray construction. Each 2.0 mm tissue core was taken from the representative region of each paraffin block. A tissue microarray containing the 35 × 4 carcinoma sample cores was constructed. Control tissues were four cancer free representative tissue samples. Cancer free tissue samples from gastric antrum and corpus could be retrieved only from 29 patients with GC. A tissue microarray containing the 29 × 4 tumor-free sample cores was constructed as well.

### 4.3. Immunohistochemical Analysis of 5-LOX, BLT1, BLT2, CysLT-1, CysLT-2

Immunohistochemical studies were performed the automated immunohistochemistry slide staining system by Ventana NexES (Ventana Medical System, Strasbourg, France). Three μm thick, formalin-fixed, paraffin-embedded serial tissue sections from all patients were deparaffinized and dehydrated. For antigen retrieval, the sections were treated by microwave heating either in 1 mM sodium citrate buffer for 5-LOX and BLT-2 (10 minutes, 600 W, pH 6.0) or in 10 mM EDTA for BLT-1 and CysLT1/2 (20 min, 450 W, pH 8.0). All sections were incubated with specific primary antibodies at 37 °C for 30 min and followed by PBS-washing (details are listed in Table 3). Positive immunohistochemical reactions were revealed using the iVIEW™ DAB Detection Kit (Ventana, Germany) as chromogen substrate. Specimens were counterstained with hematoxilin and mounted with DEPEX^®^. Specificity of immunostaining was checked by replacing the primary antibody with non-immune serum or isotype-specific irrelevant antibodies. All negative controls did not reveal any signal (data not shown). Samples were examined by two independent reviewers (M.V., C.H.).

For all antibodies immunohistochemical stainings were semi-quantitatively assessed for its staining intensity (SI; 0 = no, 1 = weak, 2 = moderate, 3 = strong staining) and for the percentage of positive cells (PP; 0 = no, 1 = < 10%, 2 = 10%–50%, 3 = 51%–80%, 4 = > 80% positive cells). For each sample an immunoreactive score (IRS) was calculated as SI × PP with a possible maximum score of 12 as described previously [18].

### 4.4. Statistical Evaluation

All data were entered into a database using the Microcal Origin™ 8.0 program package (Northhampton, MA, USA). Data are expressed as raw, median, mean ± standard deviations (sd), or 95% CI (confidence intervals), if not stated otherwise. Pairwise comparisons between data from GC and tumor-free mucosa were performed individually for each protein using Mann-Whitney U test. A two-sided p-value of <0.05 was regarded to be significant for all comparisons.

## 5. Conclusions

Here, we report for the first time an upregulation of both LTB4 receptors (BLT-1, BLT-2) and CysLT-1 in GC compared to adjacent tumor-free mucosa. Furthermore, we provide evidence that the two histological types of GC, intestinal and diffuse, partially differ regarding their expression pattern of leukotriene receptors. Whether these findings can be translated into clinically applicable approaches, e.g., new therapeutic strategies, needs to be explored in future studies.

## Figures and Tables

**Table 1.** IHC expression of 5-LOX, BLT-1, BLT-2, CysLT-1 and CysLT-2 in GC. high: Immunoreactive score (IRS) 9–12; intermediate: IRS 5–8; low: IRS 1–4.

	**High, n (%)**	**Intermediate, n (%)**	**Low, n (%)**	**Absent, n (%)**	**Overall, n (%)**
**5-LOX**	12 (34.3)	16 (45.7)	4 (11.4)	3 (8.6)	32 (91.4)
**BLT-1**	6 (17.1)	12 (34.3)	16 (45.7)	1 (2.9)	34 (97.1)
**BLT-2**	0 (0)	8 (22.9)	23 (65.7)	4 (11.4)	31 (88.6)
**CysLT-1**	19 (54.3)	13 (37.1)	2 (5.7)	1 (2.9)	34 (97.1)
**CysLT-2**	7 (20.6)	14 (41.2)	8 (23.5)	5 (14.7)	29 (85.3)

**Table 2.** Grading and staging *.

	**Intestinal type (%)**	**Diffuse type (%)**	**Mixed type (%)**
**G1**	0	0	0
**G2**	9	0	1
**G3**	13	6	5
**Stage I**	3	0	2
**Stage II**	6	0	1
**Stage III**	5	2	1
**Stage IV**	8	4	2

* Stage given by examination of surgical specimens according to the *TNM Classification of Malignant Tumours* 6th ed. [36]. Because of the small number of patients in each group, a further sub-classification of stage I in stage IA and IB as well as of stage III in stage IIIA and IIIB is not reported.

**Table 3.** Characteristics of antibodies for immunohistochemical analysis.

	**Antibody; Company; final dilution**
**5-LOX**	Rabbit polyclonal antibody 160402; Cayman Chemical, Ann Arbor, Mi, U.S.A.; dilution 1:100
**BLT-1**	Rabbit polyclonal antibody 120114; Cayman Chemical, Ann Arbor, Mi, U.S.A.; dilution 1:100
**BLT-2**	Rabbit polyclonal antibody SP4368P; Acris Antibodies, Hiddenhausen, Germany; dilution 1:25
**CysLT-1**	Rabbit polyclonal antibody SP4108P; Acris Antibodies, Hiddenhausen, Germany; dilution 1:100
**CysLT-2**	Rabbit polyclonal antibody LS-A2255; Biozol Eching, Germany; dilution 1:100
